# Patient-reported outcome measures for acute rhinosinusitis in adults and children: a systematic review of the quality of existing instruments

**DOI:** 10.1186/s12955-024-02289-0

**Published:** 2024-09-12

**Authors:** Ann-Kristin Baalmann, Sophie Nestler, Theresa Donhauser, Christian Apfelbacher, Katharina Piontek

**Affiliations:** Institute of Social Medicine and Health Systems Research, Medical Faculty, Otto-von-Guericke University Magdeburg, Leipziger Str. 44, Magdeburg, 39120 Germany

**Keywords:** Acute rhinosinusitis, Patient-reported outcome measures, COSMIN

## Abstract

**Background:**

Acute rhinosinusitis (ARS) is a self-limiting inflammation of the nose and sinuses caused by viral or bacterial infections that requires primarily symptomatic treatment. Patient-reported outcome measures (PROMs) are suitable tools for the assessment of the effectiveness of remedies for ARS from the patient’s perspective in clinical trials and real-world studies. Data regarding the quality of existing PROMs for ARS are limited.

**Purpose:**

To conduct a systematic review of the quality of existing disease-specific PROMs for use in adults and children with ARS according to the COnsensus-based Standards for the selection of health Measurement INstruments (COSMIN) methodology, and to derive recommendations for use of the identified instruments in future clinical studies.

**Methods:**

We systematically searched PubMed, Web of Science and Embase for studies reporting on the development and/or validation of PROMs for ARS. We assessed the methodological quality of each included study, evaluated the quality of measurement properties per PROM and study, and graded the evidence. Based on the overall evidence, we derived recommendations for use of the instruments.

**Results:**

We identified four studies on three PROMs measuring symptoms of ARS and quality of life in adults (Sinonasal Outcome Test-16, SNOT-16; Measurement of Acute Rhinosinusitis, MARS; Rhinosinusitis Quality-of-Life Assessment, RhinoQoL). For ARS in children, we identified two studies on two PROMs measuring symptoms of ARS (Pediatric Rhinosinusitis Symptom Scale, PRSS; Sinus Symptom Questionnaire, S5). Our assessment of measurement properties indicates that all instruments require further validation before they can be unrestrictedly recommended for use in future research (COSMIN category B). In particular, there were no content validity studies for any of the identified instruments, but also data on other important measurement properties, e.g., structural validity, are lacking.

**Conclusion:**

Currently, no PROM for ARS in adults and children can be unrestrictedly recommended based on the evaluation of their quality. Further validation is required for all identified PROMs. Content validation involving patients and experts should be given priority.

**Systematic review registration:**

OSF (10.17605/OSF.IO/VAP8U).

**Supplementary Information:**

The online version contains supplementary material available at 10.1186/s12955-024-02289-0.

## Background

Acute rhinosinusitis (ARS) is defined as an inflammation of the nose and the paranasal sinuses commonly occurring in the context of a viral cold, less often resulting from bacterial infections [1, 2]. Typical symptoms include nasal congestion, purulent nasal discharge, headache, cough, and facial pain or tenderness [3]. The condition is naturally self-limiting, and thus, treatment is aimed at reducing signs and symptoms of ARS for the comfort and well-being of the patient (symptomatic treatment). Since primarily over-the-counter medicinal products are used for self-medication [4], evaluating the effectiveness of these remedies from the patient’s perspective is of particular importance. For this purpose, patient-reported outcome measures (PROMs) are valuable tools. PROMs are standardized questionnaires for the measurement of various health outcomes directly from the patient including disease symptoms, treatment side effects, functional outcomes, and multidimensional constructs such as health-related quality of life (HRQoL) [5]. In order to select a reliable and valid PROM, the measurement properties of the instruments should be evaluated in addition to content-related and feasibility aspects. As a guideline for the selection of PROMs in research and clinical practice, the COnsensus-based Standards for the selection of health Measurement INstruments (COSMIN) methodology [6] has been developed. COSMIN offers a methodological approach with detailed, standardized and transparent criteria and practical tools for selecting the most appropriate instrument [7]. Until now, no systematic evaluation of the quality of PROMs for ARS applying the COSMIN methodology has been carried out. The present study aimed to systematically assess the quality of existing PROMs for use in adults and children with ARS, and to derive recommendations for their use in future research.

## Methods

### Protocol and registration

This systematic review was performed following the recommendations of the Preferred Reporting Items for Systematic Reviews and Meta-Analyses Protocols (PRISMA-P) statement [8], and the COSMIN guideline and manual for systematic reviews of PROMs [6, 9]. The study protocol is registered in the open registries network (https://osf.io/vap8u).

### Search strategy

A systematic search of the literature was conducted in September 2023 in the databases PubMed, Web of Science and Embase. The search strategy included the following elements:

#### Target population

Adults and children with ARS. A comprehensive compilation of controlled vocabulary and free text terms from the literature was used to enable a high level of sensitivity.

#### Construct of interest

All PROMs related to ARS were included.

#### Measurement properties

The validated and sensitive search filter for PubMed by Terwee et al. [10] was applied.

#### Feasibility of PROMs

The search terms for the concept ‘feasibility’ of Heinl et al. [11] were used.

#### Exclusion filter

Irrelevant publication types were excluded by applying the filter by Terwee et al. [10]. There were no restrictions regarding publication date and language.

For search in PubMed, the elements were combined as follows: (((A AND B AND (C OR D)) OR (C AND E)) NOT F), in words: (((population AND construct AND (measurement properties OR feasibility)) OR (measurement properties AND individual disease-specific PROMs)) NOT exclusion filter). The search syntax for PubMed is shown in Additional file 1. The syntax and index terms were adapted accordingly for the searches in Web of Science and Embase.

### Inclusion and exclusion criteria

Eligible studies addressed disease-specific PROMs for ARS. The main objective of the studies had to be the development of a PROM (“development study”) and/or the evaluation of the measurement properties of a PROM (“validation study”). Studies using PROMs only as an outcome measure or for the validation of another instrument were excluded. Furthermore, studies without available full text were excluded as abstracts provide too limited information about the design of a study. Inclusion and exclusion criteria are depicted in Table 1.

### Study selection

All records were exported to Citavi 6 for further processing. After duplicates were removed, the screening of titles, abstracts and full texts was performed using Rayyan [12]. Two independent reviewers assessed the titles and abstracts of the publications according to the inclusion and exclusion criteria to assess initial eligibility. The corresponding full texts of the articles considered suitable were also evaluated by two reviewers independently according to the predefined criteria. Any disagreements were resolved through discussions involving a third reviewer.

### Data extraction and methodological assessment

The quality of development and validation studies and the quality of the PROMs were evaluated following three sub-steps as outlined in the COSMIN manual (based on [4, 7, 11]). The measurement properties were evaluated in the following order:

1. Assessment of content validity.
2. Assessment of internal structure including structural validity, internal consistency, and cross-cultural validity/measurement invariance.
3. Assessment of the remaining measurement properties, including reliability, measurement error, criterion validity, hypothesis testing for construct validity, and responsiveness.

#### Assessment of the quality of development and validation studies

The methodological quality of each single study on a measurement property was evaluated by two reviewers independently using the COSMIN Risk of Bias checklist. This checklist consists of 10 boxes containing all standards required for the assessment of the quality of a study on that specific measurement property (Table 2). The quality of each study was rated on a 4-point rating scale as either very good, adequate, doubtful or inadequate. The overall quality of a study was determined by the lowest rating of any standard in the box (“worst score counts”).

Content validity is considered the most important measurement property and was evaluated by assessing the available evidence from content validity and PROM development studies. If the PROM development study was rated as “inadequate” and no content validity studies were available, or if only content validity studies of inadequate quality were available, content validity was rated by the reviewers. In these cases, the reviewers’ rating determined the overall rating. Reviewers’ ratings are based on sighting of the questionnaires followed by a discussion of two independent reviewers to find consensus [13].

In addition to data on measurement properties, data on characteristics of the included PROMs and study populations, as well as data on interpretability and feasibility were extracted.

#### Assessment of the quality of measurement properties

The results of each single study on a measurement property were rated against the criteria for good measurement properties as either sufficient (+), insufficient (-), or indeterminate (?) (Table 3).

#### Grading of the quality of evidence and recommendation

The quality of evidence was summarized per measurement property and PROM and also evaluated according to the criteria for good measurement properties. The quality of evidence was graded using the Grading of Recommendations Assessment, Development and Evaluation (GRADE) approach considering the methodological quality of studies, total sample size, and consistency of results as either high, moderate, low, or very low [14]. No grading of evidence was conducted if the overall ratings were indeterminate or inconsistent.

To generate recommendations for use of the identified PROMs in future clinical studies, each instrument was categorized according to its methodological quality following the recommendations of the COSMIN group [6]:

A. PROMs with evidence for sufficient content validity (any level) and at least low-quality evidence for sufficient internal consistency that can be recommended for use as results obtained from these measures are considered trustworthy.
B. PROMs categorized not in A or C that have the potential to be recommended for use but require further validation.
C. PROMs with high-quality evidence for an insufficient measurement property that should not be recommended for use.

If only category B PROMs are available, the PROM with the best evidence for content validity can be preliminarily recommended for use until further evidence is given [14].

## Results

### Literature search

**Fig. 1:**
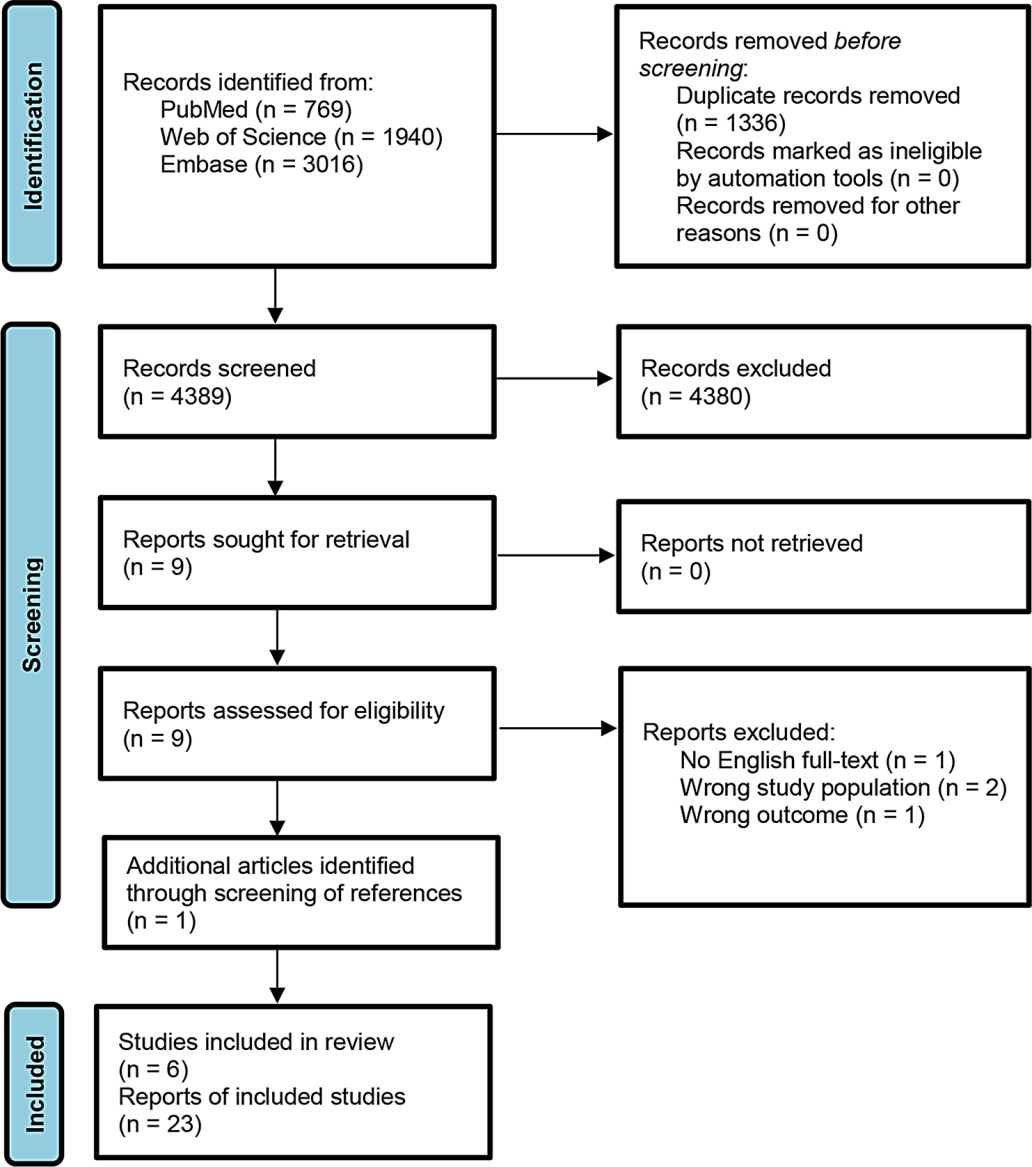
Flow diagram according to the Preferred Reporting Items for Systematic reviews and Meta-Analyses (PRISMA) guidelines

The search resulted in a total of 4,389 records without duplicates (Fig. 1). Based on title and abstract, 4,380 records were excluded. Nine full-text articles were assessed for eligibility of which four were excluded. Screening of the references of the included studies and searching Google Scholar yielded one additional relevant result. For data extraction, six studies reporting on five PROMs were included. For adults with ARS, we identified two studies reporting on the Sinonasal Outcome Test-16 (SNOT-16) [15, 16], and one study reporting on the Measurement of Acute Rhinosinusitis (MARS) [17]. Further, handsearching revealed a doctoral thesis aiming to validate the German version of the Rhinosinusitis Quality-of-Life Assessment (RhinoQoL) [18]. For comprehensiveness, we additionally searched the development study of the RhinoQoL [19], and included it in our analyses. We further identified two studies reporting on two PROMs for children with ARS (Pediatric Rhinosinusitis Symptom Scale, PRSS [20]; Sinus Symptom Questionnaire, S5 [21]).

### Characteristics of instruments and study populations

Details of the included PROMs and study populations are displayed in Tables 4 and 5. The purpose of the SNOT-16, MARS and RhinoQoL is to measure ARS symptoms and quality of life in adults. The PRSS was designed as a proxy-reported outcome measure for the assessment of disease symptoms in children with ARS aged 2 to 12 years to be completed by their parents. The S5 is also a proxy-reported outcome measure of ARS symptoms in children to be completed by their parents, but without an age limit. Notably, the identified instruments have varying recall periods including two weeks (SNOT-16), seven days (RhinoQoL), 24 h (PRSS) and the last few days (S5). No recall period is reported for the MARS. The sample sizes of the development and validation studies ranged from 81 to 1611 participants. The average age in studies validating PROMs for adult patients ranged from 22.8 to 40.4 years. In the studies on the PRSS, the mean age of the children ranged from 5.6 (validation study) to 6.4 years (development study) [20]. Age distribution of participants in the study on the S5 was as follows: 46% were younger than 6 years, 26% were between 6 and 12 years old, and 27% were older than 12 years.

### Interpretability and feasibility

Regarding PROMs for adults, anchor-based minimal important difference (MID) estimates were provided for the SNOT-16 for interpretability. In the validation study of Garbutt et al. [15], the mean change in SNOT-16 score ranged from 0.48 unit at day 3 to 0.80 unit at day 10 in participants reporting a small change, and from 0.8 unit at day 3 to 1.3 units at day 10 in individuals reporting a large change in symptoms (score range: 0–3). Considering the total score, a MID of 13.56 (score range: 0–48) has been reported in the validation study of Quadri et al. [16]. Additionally, no floor or ceiling effects and no significant skew of the data were reported in this study [16]. For the MARS and RhinoQoL, no information on interpretability were available. Concerning PROMs for children, the S5 showed a sensitivity of 58% and a specificity of 95%. Ceiling effects were reported for the PRSS total score, showing that 0.5–0.8% of children reached the highest possible score. Also, an MID estimate of 3.0 was provided [20].

With respect to the feasibility of PROMs for adults, it was reported that the SNOT-16 was easy to use and took less than five minutes to complete. Additionally, the instrument was well accepted by the patients, and its use did not require a special training. Furthermore, while the validation study by Garbutt et al. reported no missing data [15], a minimal amount of missing data at baseline and a higher proportion at test of cure was reported in the validation study by Quadri et al. [16]. The MARS was reported to take less than three minutes to complete [17], indicating that this instrument is very economic. There was no information on feasibility aspects for the RhinoQoL for adults and for the S5 and PRSS for children. All PROMs for adults are self-administered. The PROMs for children are completed by parents. Information on access to all identified PROMs is given in Additional file 2.

### Measurement properties of identified PROMs

#### Content validity

The PROM development studies of the SNOT-16, MARS, RhinoQoL and S5 were rated “inadequate” since the instruments were not developed in a sample representing the target population. The development study of the PRSS received a “doubtful” rating because the development of the instrument was based solely on a quantitative survey with an inappropriate sample size. The overall ratings for content validity according to the COSMIN Risk of Bias checklist are presented in Additional file 3. Notably, all instruments were initially designed for use in individuals with chronic sinusitis and adapted for ARS in the identified validation studies. Since no content validity studies were available for the included instruments, the content validity ratings are based on the reviewers’ evaluation, which results in very low quality of evidence. The reviewers rated the content validity of the SNOT-16, MARS, RhinoQoL, PRSS and S5 as sufficient. The results of the content validity assessment is detailed in Additional file 4.

#### Remaining measurement properties

The results of the assessment of the quality of the studies on these measurement properties and the rating of the methodological quality of the included PROMs are presented in Table 6. Based on the six available validation studies, we assessed the methodological quality of 23 single studies on measurement properties including structural validity, internal consistency, test-retest reliability, hypotheses testing for construct validity and responsiveness. None of the included studies tested for cross-cultural validity, measurement invariance or criterion validity.

#### Summary of findings and grading of the quality of evidence

A summary of the results per measurement property and PROM as well as the graded quality of evidence is displayed in Table 7.

#### Recommendations for further use

All included PROMs were classified in category B (Table 8), indicating that they have the potential to be recommended for use but require further validation.

## Discussion

In our synthesized evaluation of the quality of PROMs for adults and children with ARS following the COSMIN methodology we identified three instruments for use in adults and two instruments for use in children, all of which require further validation before they can be unrestrictedly recommended for use in future clinical studies. Content validity is a major weakness of the available instruments, but also data on other important measurement properties, e.g., structural validity, are lacking.

A comprehensive literature search in three large databases was conducted using a search strategy based on validated search filters and with few restrictions. Screening of the references of included studies and searching Google Scholar extended the search and allowed further potentially relevant studies to be identified. Moreover, the COSMIN and PRISMA standards were adhered to ensure high-quality evidence synthesis. By involving three independent reviewers for the assessment of risk of bias and data extraction, a particularly thorough discussion on the identified studies and PROMs ensued. Although the focus on disease-specific measurement instruments allows a particularly precise investigation of the patient perspective, the inclusion of generic instruments might also have generated interesting findings and evidence.

The recommendation for use of a PROM according to the guidelines of the COSMIN group is based on the evaluation of content validity and structural validity. We found sufficient content validity for all included instruments, but this was solely rated by the reviewers, resulting in very low quality of evidence. Importantly, SNOT-16 and RhinoQoL were initially designed for use in chronic rhinosinusitis and adapted for ARS in validation studies. The SNOT-16 was derived from the SNOT-20 [22, 23], which is a modified version of the Rhinosinusitis Outcome Measure-31 (RSOM-31) [24]. There are also other versions available, primarily for use in patients with chronic rhinosinusitis, such as the SNOT-22 [25] that represents another modification of the SNOT-20 based on expert focus group discussions, or the SNOT-25 [26], which is a modification of the SNOT-22 containing three new items derived from patient interviews and literature searches. Content validation involving patients and experts from different disciplines should be given priority for all identified PROMs for use in patients with ARS. In these evaluations, the content of PROMs for chronic rhinosinusitis should be examined regarding their relevance for ARS and the construct to be measured such as symptoms and quality of life. The comprehensiveness of the questionnaire for the target group should also be re-assessed. Issues regarding the appropriateness of the recall periods of the instruments occurred during reviewers’ rating. For example, SNOT-16 and RhinoQoL assessments refer to the last 14 and 7 days, respectively. For use of the instruments in patients with ARS, recall periods should be adapted according to the course of the disease, e.g., referring to the past 12–24 h.

According to COSMIN, structural validity is a prerequisite for interpreting analyses of internal consistency [9]. Although results on internal consistency are reported for the SNOT-16, RhinoQoL, MARS and PRSS, they cannot be correctly interpreted due to missing data on structural validity. Future studies should thus contain analyses on structural validity.

For the SNOT-16, the PRSS and the S5, data on test-retest-reliability were available resulting in a sufficient rating according to the criteria for good measurement properties. However, different time intervals were used limiting the comparability of the presented results. These findings indicate the need to determine appropriate time intervals for assessing test-retest reliability in patients suffering from ARS, such as a daily assessment until symptom remission.

Regarding PROMs for adults, no instruments showed significant evidence for insufficient interpretability. For the SNOT-16, only evidence for very minimal floor and ceiling effects and no significant skew of the data were reported. Also, the PROMs for children did not show any signs of insufficient interpretability. For the S5, acceptable values for sensitivity and specificity were calculated and for the PRSS, only minimal ceiling effects were reported. With respect to feasibility aspects, no substantial missing data were reported. In particular, SNOT-16 and MARS seem to be very feasible and economic due to the very short completion times. It should be noted that no information on the interpretability of MARS and RhinoQoL, and no information on the feasibility of RhinoQoL, the S5 and PRSS were available.

## Conclusion

Three PROMs for use in adults and two PROMs for use in children with ARS were identified. All included instruments can potentially be recommended only after further validation. Future studies should focus on content validation and on analyses of structural validity and internal consistency of existing PROMs.

**Table 1:** Inclusion and exclusion criteria

	Inclusion criteria	Exclusion criteria
Population	Adults and children with acute rhinosinusitis	Chronic rhinosinusitis, allergic rhinosinusitis, allergic rhinitis
Study design	PROM development and/or validation study	All other study designs
Outcome	All patient-reported and proxy-reported outcomes	Non patient-reported outcomes, e.g. biomarkers, laboratory data
Type of measurement instrument	Patient-reported outcome measurement instruments	All others
Publication type	Articles with available full text	Abstracts

*PROM* patient-reported outcome measure

**Table 2:** Boxes of the COSMIN risk of Bias checklist

**Content validity**	
Box 1	PROM development
Box 2	Content validity
**Internal structure**	
Box 3	Structural validity
Box 4	Internal consistency
Box 5	Cross-cultural validity/measurement invariance
**Remaining measurement properties**	
Box 6	Reliability
Box 7	Measurement error
Box 8	Criterion validity
Box 9	Hypotheses testing for construct validity
Box 10	Responsiveness

*COSMIN* Consensus-based Standards for the selection of health Measurement Instruments, *PROM* patient-reported outcome measure

**Table 3:** Criteria for good measurement properties

Measurement property	Rating	Criteria
Structural validity	+	**CTT** CFA: CFI or comparable measure > 0.95 OR RMSEA < 0.06 OR SRMR < 0.08^a^ **IRT/Rasch** No violation of *unidimensionality*^b^: CFI or TLI or comparable measure > 0.95 OR RMSEA < 0.06 OR SRMR < 0.08 *AND* no violation of *local independence*: residual correlations among the items after controlling for the dominant factor < 0.20 OR Q3’s < 0.37 *AND* no violation of *monotonicity*: adequate looking graphs OR item scalability > 0.30 *AND* adequate *model fit* IRT: χ^2^ > 0.001 Rasch: infit and outfit mean squares ≥ 0.5 and ≤ 1.5 OR Z-standardized values > -2 and < 2
	?	CTT: not all information for ‘+’ reported IRT/Rasch: model fit not reported
	-	Criteria for ‘+’ not met
Internal consistency	+	At least low evidence^c^ for sufficient structural validity^d^” AND Cronbach’s alpha(s) ≥ 0.70 for each unidimensional scale or subscale^e^
	?	Criteria for “At least low evidence^c^ for sufficient structural validity^d^” not met
	-	At least low evidence^c^ for sufficient structural validity^d^ and Cronbach’s alpha(s) < 0.70 for each unidimensional scale or subscale^e^
Reliability	+	ICC or weighted Kappa ≥ 0.70
	?	ICC or weighted Kappa not reported
	-	ICC or weighted Kappa < 0.70
Measurement error	+	SDC or LoA < MIC^d^
	?	MIC not defined
	-	SDC or LoA > MIC
Hypotheses testing for construct validity	+	The result is in accordance with the hypothesis^f^
	?	No hypothesis defined (by the review team)
	-	The result is not in accordance with the hypothesis^f^
Cross-cultural validity/measurement invariance	+	No important differences found between group factors (such as age, gender, language) in multiple group factor analysis OR no important DIF for group factors (McFadden’s R^2^ < 0.02)
	?	No multiple group factor analysis OR DIF analysis performed
	-	Important differences between group factors OR DIF was found
Criterion validity	+	Correlation with gold standard ≥ 0.70 OR AUC ≥ 0.70
	?	Not all information for ‘+’ reported
	-	Correlation with gold standard < 0.70 OR AUC < 0.70
Responsiveness	+	The result is in accordance with the hypothesis^f^ OR AUC ≥ 0.70
	?	No hypothesis defined (by the review team)
	-	The result is not in accordance with the hypothesis^f^ OR AUC < 0.70

The criteria are based on Terwee et al. and Prinsen et al.

*AUC* area under the curve, *CFA* confirmatory factor analysis, *CFI* comparative fit index, *CTT* classical test theory, *DIF* differential item functioning, *ICC* intraclass correlation coefficient, *IRT* Item response theory, *LoA* limits of agreement, *MIC* minimal important change, *RMSEA* root mean square error of approximation, *SEM* standard error of measurement, *SDC* smallest detectable change, *SRMR* standardized root mean residuals, *TLI* Tucker-Lewis index

“+” = sufficient, “-“ = insufficient, “?” = indeterminate

^a^To rate the quality of the summary score, the factor structure should be equal across studies

^b^Unidimensionality refers to a factor analysis per subscale, while structural validity refers to a factor analysis of a (multidimensional) patient-reported outcome measure

^c^As defined by grading the evidence according to the GRADE approach

^d^This evidence may come from different studies

^e^The criteria ‘Cronbach’s alpha < 0.95’ was deleted, as this is relevant in the development phase of a PROM and not when evaluating an existing PROM

^f^The results of all studies should be taken together and it should then be decided if 75% of the results are in accordance with the hypotheses

**Table 4:** Characteristics of the included instruments

	PROMs for use in adults			PROMs for use in children	
	Sinonasal Outcome Test-16 (SNOT-16)	Measurement of Acute Rhinosinusitis (MARS)	Rhinosinusitis Quality of Life Questionnaire (RhinoQoL)	Pediatric Rhinosinusitis Symptom Scale (PRSS)	Sinus Symptom Questionnaire (S5)
Construct	Quality of life (including symptoms)	Quality of life (including symptoms)	Quality of life (including symptoms)	Symptoms	Symptoms
Target population	Adult patients with acute or chronic rhinosinusitis	Adult patients with acute rhinosinusitis	Adult patients with acute or chronic rhinosinusitis	Young children (2–12 years) with acute rhinosinusitis ◊ Parents (proxy-reported outcome measure)	Children with acute rhinosinusitis ◊ Parents (proxy-reported outcome measure)
Recall period	2 weeks	Present (?)	7 days	24 h	Last few days
(Sub)scales (number of items)	0 subscales (16 items)	0 subscales (13 items)	3 subscales (Symptom frequency, symptom bothersomeness, symptom impact); 14 items	0 subscales (8 items)	0 subscales (5 items)
Response options and range of scores/scoring	0 to 3 (no problem; mild problem; moderate problem; severe problem); score 0–48 (sum of all items)	0 to 3 (no problem; mild problem; moderate problem; severe problem); score 0–39 (sum of all items)	Various response options: yes/no, 1 to 5 (none of the time, a little of the time, some of the time, most of the time, all of the time), 0 (not bothered at all) to 10 (bothered a lot)	no, almost none, a little, some, a lot, an extreme amount; scoring not reported	Item A-D: 0 to 3 (not present; small problem; medium problem; large problem) and don’t know; Item E: 0 (none, clear), 3 (yellow, green), don’t know; score 0–15 (sum of all items)
Available translations	English + 90 translations (including German)	Czech + English	English, German, French, Persian	English	English

*PROM* patient-reported oucome measure

**Table 5:** Characteristics of the included study populations

PROM	Reference	Sample size	Age mean (SD) or median in years	Setting	Country (Language)	Measurement properties
**Quality of life**						
**SNOT-16**	Garbutt et al. (2011)	*N* = 166	32 (range 18–69)	Primary care practices	USA (English)	Internal consistency, test-retest reliability, construct validity, responsiveness
	Quadri et al. (2013)	*N* = 347	Treatment arm: 40.1 (13.8); Placebo arm: 40.3 (13.0)	Clinical sites	USA (English)	Internal consistency, construct validity, responsiveness
**MARS**	Hornáčková et al. (2014)	*N* = 100	Patient group: 40.4 (range 18–71); Control group: 22.8	Ears, nose, throat offices and outpatient department of a university hospital	Czech Republic (Czech)	PROM development, internal consistency, construct validity, responsiveness
**RhinoQoL**	Petrat (2020)	*N* = 81	≥ 18 years	Clinical site	Germany (German)	Internal consistency, construct validity, responsiveness
**Symptoms**						
**PRSS**	Shaikh et al. (2019)	Development: *N* = 258; Validation: *N* = 185	Development: 6.4 (2.9), Validation: 5.6 (2.7)	Ambulatory pediatric clinics	USA (English)	PROM development, structural validity, internal consistency, test-retest reliability, responsiveness
**S5**	Garbutt et al. (1999)	Development: *N* = 1611; Validation: *N* = 93	46% <6 years; 26% 6–12 years; 27% >12 years	Community pediatric ambulatory care practice	USA (English)	PROM development, test-retest reliability, responsiveness

*PROM* patient-reported oucome measure, *MARS* Measurement of Acute Rhinosinusitis, *PRSS* Pediatric Rhinosinusitis Symptom Score, *RhinoQoL* Rhinosinusitis Quality-of-Life Questionnaire, *SNOT-16* Sinonasal Outcome Test-16, *S5* Sinusitis Symptom Questionnaire

**Table 6:** Quality of studies on measurement properties and methodological rating of the instruments

PROM	Reference	Methodological quality (rating^1,2^)					
		Structural validity	Internal consistency	Test-Retest-Reliability	Construct validity (Comparator instrument)	Construct validity (Known-groups)	Responsiveness
SNOT-16	Garbutt et al. 2011	-	Very good (?)	Doubtful (+)	-	Very good (±)	Very good (+)
	Quadri et al. 2013	-	Doubtful (?)	-	Adequate (+)	-	Very good (+)
MARS	Hornáčková et al. 2014	-	Doubtful (?)	-	-	Doubtful (+)	Very good (+)
RhinoQoL	Atlas et al. 2005*	-	Doubtful (?)	-	Adequate (±)	Doubtful (±)	-
	Petrat 2020	-	Doubtful (?)	-	Adequate (+)	-	Comparator instrument: Adequate (-)
							Known-groups: Doubtful (+)
PRSS	Shaikh et al. 2019	Adequate (?)	Doubtful (?)	Adequate (+)	-	-	Doubtful (+)
S5	Garbutt et al. 1999	-	-	Doubtful (+)	-	-	Inadequate (±)

*PROM* Patient-reported outcome measure; *MARS* Measurement of Acute Rhinosinusitis, *PRSS* Pediatric Rhinosinusitis Symptom Score, *RhinoQoL* Rhinosinusitis Quality-of-Life Questionnaire, *SNOT-16* Sinonasal Outcome Test-16, *S5* Sinusitis Symptom Questionnaire

^1^No study has analyzed cross-cultural validity/measurement invariance, measurement error and criterion validity

^2^Rating: *(+)* sufficient, *(-)* insufficient, *(?)* indeterminate, (±) inconsistent

* The development study of the RhinoQoL was additionally searched and included for comprehensive assessment

**Table 7:** Summary of findings

PROM/Measurement property	Summary or pooled result	Overall rating	Quality of evidence
**Quality of life (including symptoms)**			
***Sinonasal Outcome Test-16 (SNOT-16)***			
Internal consistency	Alpha = 0.82; sample size = 166; alpha = 0.874 ; sample size: 374; no evidence for sufficient structural validity	Indeterminate	-
Test-retest-reliability	ICC = 0.73; sample size: 166	Sufficient	Low (due to risk of bias)
Construct validity (comparator instruments)	6 out of 8 hypotheses confirmed; sample size: 374	Sufficient	High
Construct validity (known-groups)	3 out of 6 hypotheses confirmed; sample size: 166	Inconsistent	-
Responsiveness	1 of 1 hypothesis confirmed; sample size: 374	Sufficient	High
***Measurement of Acute Rhinosinusitis (MARS)***			
Internal consistency	Alpha = 0.679; no evidence for sufficient structural validity; sample size: 50	Indeterminate	-
Construct validity (known-groups)	1 of 1 hypothesis confirmed; sample size: 100	Sufficient	Low (due to risk of bias)
Responsiveness	1 of 1 hypothesis confirmed; sample size: 50	Sufficient	High
***Rhinosinusitis Quality of Life Questionnaire (RhinoQoL)***			
Internal consistency	Alpha = 0.75; sample size = 81; alpha_frequency_ = 0.45, alpha_bothersomeness_ = 0.28, alpha_impact_ = 0.85; sample size: 47; no evidence for sufficient structural validity	Indeterminate	-
Construct validity (comparator instruments)	10 out of 12 hypotheses confirmed; sample size: 128	Sufficient	High
Construct validity (known-groups validity)	2 out of 3 hypotheses confirmed; sample size: 47	Inconsistent	Very low (due to risk of bias and imprecision)
Responsiveness (comparator instrument)	1 of 1 hypothesis not confirmed; sample size: 81	Insufficient	Low (due to risk of bias and imprecision)
Responsiveness (known-groups)	3 out of 4 hypotheses confirmed; sample size: 81	Sufficient	Very low (due to risk of bias and imprecision)
***Symptoms***			
***Pediatric Rhinosinusitis Symptom Scale (PRSS)***			
Structural validity	Not reported; sample size: 185	Indeterminate	-
Internal consistency	Alpha = 0.79; no evidence for sufficient structural validity; sample size: 185	Indeterminate	-
Test-retest-reliability	ICC = 0.75; sample size: 185	Sufficient	Moderate (due to risk of bias)
Responsiveness	2 out of 2 hypotheses confirmed; sample size: 185	Sufficient	Low (due to risk of bias)
***Sinus Symptom Questionnaire (S5)***			
Test-retest-reliability	ICC = 0.94; sample size: 26	Sufficient	Low (due to risk of bias)
Responsiveness	2 out of 3 hypotheses confirmed; sample size: 29–31	Inconsistent	-

*ICC* intraclass correlation coefficient, *PROM* patient-reported outcome measure

**Table 8:** Recommendations for use

	Category A			Category C		
PROM	Sufficient content validity (any level)	At least low quality evidence for sufficient internal consistency		High quality evidence for an insufficient measurement property		Recommendation
SNOT-16	Yes	No		No		B
MARS	Yes	No		No		B
RhinoQoL	Yes	No		No		B
PRSS	Yes	No		No		B
S5	Yes	No		No		B

*B* COSMIN category B, *PROM* Patient-reported outcome measure; *MARS* Measurement of Acute Rhinosinusitis, *PRSS* Pediatric Rhinosinusitis Symptom Score, *RhinoQoL* Rhinosinusitis Quality-of-Life Questionnaire, *SNOT-16* Sinonasal Outcome Test-16, *S5* Sinusitis Symptom Questionnaire, Yes fulfilled, No not fulfilled

## Electronic supplementary material

Below is the link to the electronic supplementary material.

Supplementary Material 1

Supplementary Material 2

Supplementary Material 3

Supplementary Material 4

## Data Availability

No datasets were generated or analysed during the current study.

## Abbreviations

ARS: Acute rhinosinusitis
COSMIN: COnsensus-based Standards for the selection of health Measurement INstruments
GRADE: Grading of Recommendations Assessment, Development and Evaluation
HRQoL: Health-related quality of life
MARS: Measurement of Acute Rhinosinusitis
MID: Minimal important difference
PRISMA-P: Preferred Reporting Items for Systematic Reviews and Meta-Analyses Protocols
PROM: Patient-reported outcome measure
PRSS: Pediatric Rhinosinusitis Symptom Scale
RhinoQoL: Rhinosinusitis Quality of Life Assessment
RSOM-31: Rhinosinusitis Outcome Measure-31
SNOT-16: Sinonasal Outcome Test-16
SNOT-20: Sinonasal Outcome Test-20
SNOT-22: Sinonasal Outcome Test-22
SNOT-25: Sinonasal Outcome Test-25
S5: Sinus Symptom Questionnaire
