# Supplementary material for: Patient-reported outcome measures for acute rhinosinusitis in adults and children: a systematic review of the quality of existing instruments
Source: Health Qual Life Outcomes. 2024 Sep 12;22:79. doi: 10.1186/s12955-024-02289-0 (PMC11395909; doi:10.1186/s12955-024-02289-0)
Supplement: Supplementary file 1 — Supplementary Material 1 [file 12955_2024_2289_MOESM1_ESM.docx]

**Additional file 2** Availability of the identified instruments

**Measurement of Acute Rhinosinusitis (MARS)**

The English version of the MARS is available as appendix of the research paper:

http://b-ent.be/Content/files/sayilar/30/2014-10-3-209-Hornackova.pdf

**Pediatric Rhinosinusitis Symptom Scale (PRSS)**

The English version of the PRSS is available as appendix of the research paper: https://doi.org/10.1016/j.jpeds.2018.11.016

**Rhinosinusitis Quality of Life Assessment (RhinoQoL)**

The English version of the RhinoQoL is available as appendix of the research paper: https://doi.org/10.1177/0194599811400686

**Sinusitis Symptom Questionnaire (S5)**

The English version of the S5 is available as appendix of the research paper:

https://doi.org/10.1023/A:1008883508462

**Sinonasal Outcome Test-16 (SNOT-16)**

The SNOT-16 is available at Washington University St. Louis for different purposes:

https://sinonasaltest.wustl.edu/licensing-snot/

A list of translations is also provided by the Washington University St. Louis:

https://sinonasaltest.wustl.edu/sino-nasal-outcome-test-snot/available-translations-2/
