# Supplementary material for: Patient-reported outcome measures for acute rhinosinusitis in adults and children: a systematic review of the quality of existing instruments
Source: Health Qual Life Outcomes. 2024 Sep 12;22:79. doi: 10.1186/s12955-024-02289-0 (PMC11395909; doi:10.1186/s12955-024-02289-0)
Supplement: Supplementary file 2 — Supplementary Material 2 [file 12955_2024_2289_MOESM2_ESM.docx]

**Additional file 4** Content validity rating of the included instruments

|  |  | **Relevance** | **Comprehensiveness** | **Comprehensibility** | **Content validity rating** |
| --- | --- | --- | --- | --- | --- |
| **Quality of life** | |  |  |  |  |
| SNOT-16 | Overall rating | + | + | + | Sufficient (+) |
|  | Quality of evidence | Very low (only reviewers rating) | | | |
|  |  |  |  |  |  |
| MARS | Overall rating: Symptom Severity | + | + | ± | Sufficient (+) |
|  | Quality of evidence | Very low (only reviewers rating) | | | |
|  |  |  |  |  |  |
| RhinoQoL | Overall rating | + | + | + | Sufficient (+) |
|  | Quality of evidence | Very low (only reviewers rating) | | | |
| **Symptoms** | |  |  |  |  |
| PRSS | Overall rating: Symptom Severity | + | + | + | Sufficient (+) |
|  | Quality of evidence | Low | | | |
|  |  |  |  |  |  |
| S5 | Overall rating | + | + | + | Sufficient (+) |
|  | Quality of evidence | Very low (only reviewers rating) | | | |

*MARS* Measurement of Acute Rhinosinusitis, *PRSS* Pediatric Rhinosinusitis Symptom Score, *RhinoQoL* Rhinosinusitis Quality-of-Life Questionnaire, *SNOT-16* Sinonasal Outcome Test-16, *S5* Sinusitis Symptom Questionnaire, *+* Sufficient, *±* Inconsistent
