# Supplementary material for: Patient-reported outcome measures for acute rhinosinusitis in adults and children: a systematic review of the quality of existing instruments
Source: Health Qual Life Outcomes. 2024 Sep 12;22:79. doi: 10.1186/s12955-024-02289-0 (PMC11395909; doi:10.1186/s12955-024-02289-0)
Supplement: Supplementary file 3 — Supplementary Material 3 [file 12955_2024_2289_MOESM3_ESM.docx]

**Additional file 1** Search strategy for PUBMED

**### A: Target population – Adults and children with acute rhinosinusitis**

"acute rhinosinusitis" [Title/Abstract] OR "acute sinusitis" [Title/Abstract] OR "upper respiratory tract infection" [Title/Abstract] OR "common cold" [Title/Abstract]

**### B: Construct – PROMs including Quality of Life**

"Patient Reported Outcome Measures"[Mesh] OR "Quality of Life"[Mesh] OR prom[tiab] OR proms[tiab] OR pro[tiab] OR pros[tiab] OR HRQL[tiab] OR HRQoL[tiab] OR QL[tiab] OR QoL[tiab] OR quality of life[tiab] OR life quality[tiab] OR health index*[tiab] OR health indices[tiab] OR health profile*[tiab] OR health status[tw] OR ((patient[tiab] OR self[tiab] OR child[tiab] OR parent[tiab] OR carer[tiab] OR proxy[tiab]) AND ((report[tiab] OR reported[tiab] OR reporting[tiab]) OR (rated[tiab] OR rating[tiab] OR ratings[tiab]) OR based[tiab] OR (assessed[tiab] OR assessment[tiab] OR assessments[tiab]))) OR ((disability[tiab] OR function[tiab] OR functional[tiab] OR functions[tiab] OR subjective[tiab] OR utility[tiab] OR utilities[tiab] OR wellbeing[tiab] OR well being[tiab]) AND (outcome[tiab] OR outcomes[tiab] OR index[tiab] OR indices[tiab] OR instrument[tiab] OR instruments[tiab] OR measure[tiab] OR measures[tiab] OR questionnaire[tiab] OR questionnaires[tiab] OR profile[tiab] OR profiles[tiab] OR scale[tiab] OR scales[tiab] OR score[tiab] OR scores[tiab] OR status[tiab] OR survey[tiab] OR surveys[tiab]))

**### C: Measurement properties**

(instrumentation[sh] OR methods[sh] OR Comparative Study[pt] OR "psychometrics" [MeSH] OR psychometr*[tiab] OR clinimetr*[tw] OR clinometr*[tw] OR outcome assessment[tiab] OR outcome measure*[tw] OR "observer variation" [MeSH] OR observer variation[tiab] OR "Health Status Indicators" [Mesh] OR "reproducibility of results" [MeSH] OR reproducib*[tiab] OR "discriminant analysis" [MeSH] OR reliab*[tiab] OR unreliab*[tiab] OR valid*[tiab] OR coefficient[tiab] OR homogeneity[tiab] OR homogeneous[tiab] OR "internal consistency" [tiab] OR (cronbach*[tiab] AND (alpha[tiab] OR alphas[tiab])) OR (item[tiab] AND (correlation*[tiab] OR selection*[tiab] OR reduction*[tiab])) OR agreement[tiab] OR precision[tiab] OR imprecision[tiab] OR "precise values" [tiab] OR test–retest[tiab] OR (test[tiab] AND retest[tiab]) OR (reliab*[tiab] AND (test[tiab] OR retest[tiab])) OR stability[tiab] OR interrater[tiab] OR inter-rater[tiab] OR intrarater[tiab] OR intra-rater[tiab] OR intertester[tiab] OR inter-tester[tiab] OR intratester[tiab] OR intra-tester[tiab] OR interobserver[tiab] OR inter-observer[tiab] OR intraobserver[tiab] OR intra-observer[tiab] OR intertechnician[tiab] OR inter-technician[tiab] OR intratechnician[tiab] OR intra-technician[tiab] OR interexaminer[tiab] OR inter-examiner[tiab] OR intraexaminer[tiab] OR intra-examiner[tiab] OR interassay[tiab] OR inter-assay[tiab] OR intraassay[tiab] OR intra-assay[tiab] OR interindividual[tiab] OR inter-individual[tiab] OR intraindividual[tiab] OR intra-individual[tiab] OR interparticipant[tiab] OR inter-participant[tiab] OR intraparticipant[tiab] OR intra-participant[tiab] OR kappa[tiab] OR kappa’s[tiab] OR kappas[tiab] OR repeatab*[tiab] OR ((replicab*[tiab] OR repeated[tiab]) AND (measure[tiab] OR measures[tiab] OR findings[tiab] OR result[tiab] OR results[tiab] OR test[tiab] OR tests[tiab])) OR generaliza*[tiab] OR generalisa*[tiab] OR concordance[tiab] OR (intraclass[tiab] AND correlation*[tiab]) OR discriminative[tiab] OR "known group" [tiab] OR factor analysis[tiab] OR factor analyses[tiab] OR dimension*[tiab] OR subscale*[tiab] OR (multitrait[tiab] AND scaling[tiab] AND (analysis[tiab] OR analyses[tiab])) OR item discriminant[tiab] OR interscale correlation*[tiab] OR error[tiab] OR errors[tiab] OR "individual variability" [tiab] OR (variability[tiab] AND (analysis[tiab] OR values[tiab])) OR (uncertainty[tiab] AND (measurement[tiab] OR measuring[tiab])) OR "standard error of measurement" [tiab] OR sensitiv*[tiab] OR responsive*[tiab] OR ((minimal[tiab] OR minimally[tiab] OR clinical[tiab] OR clinically[tiab]) AND (important[tiab] OR significant[tiab] OR detectable[tiab]) AND (change[tiab] OR difference[tiab])) OR (small*[tiab] AND (real[tiab] OR detectable[tiab]) AND (change[tiab] OR difference[tiab])) OR meaningful change[tiab] OR "ceiling effect" [tiab] OR "floor effect" [tiab] OR "Item response model" [tiab] OR IRT[tiab] OR Rasch[tiab] OR "Differential item functioning" [tiab] OR DIF[tiab] OR "computer adaptive testing" [tiab] OR "item bank" [tiab] OR "cross-cultural equivalence" [tiab])

**### D: Feasibility of PROMs**

((accepta*[Title/Abstract]) OR ("ease of use"[Title/Abstract]) OR (practica*[Title/Abstract]) OR (feasib*[Title/Abstract]))

**### E: Individual disease-specific PROMs**

“Modified Sino-Nasal Outcome Test-16” [Title/Abstract] OR “Modified Sinonasal Outcome Test-16” [Title/Abstract] OR “SNOT-16” [Title/Abstract] OR “Measurement of Acute Rhinosinusitis” [Title/Abstract]

**### F: Exclusion filter**

(‘‘addresses’’[Publication Type] OR ‘‘biography’’[Publication Type] OR ‘‘case reports’’[Publication Type] OR ‘‘comment’’[Publication Type] OR ‘‘directory’’[Publication Type] OR ‘‘editorial’’[Publication Type] OR ‘‘festschrift’’[Publication Type] OR ‘‘interview’’[Publication Type] OR ‘‘lectures’’[Publication Type] OR ‘‘legal cases’’[Publication Type] OR ‘‘legislation’’[Publication Type] OR ‘‘news’’[Publication Type] OR ‘‘newspaper article’’[Publication Type] OR ‘‘patient education handout’’[Publication Type] OR ‘‘popular works’’[Publication Type] OR ‘‘congresses’’ [Publication Type] OR ‘‘consensus development conference’’[Publication Type] OR ‘‘consensus development conference, nih’’[Publication Type] OR ‘‘practice guideline’’[Publication Type]) NOT (‘‘animals’’[MeSH Terms] NOT ‘‘humans’’[MeSH Terms])
