# Supplementary material for: Patient-reported outcome measures for acute rhinosinusitis in adults and children: a systematic review of the quality of existing instruments
Source: Health Qual Life Outcomes. 2024 Sep 12;22:79. doi: 10.1186/s12955-024-02289-0 (PMC11395909; doi:10.1186/s12955-024-02289-0)
Supplement: Supplementary file 4 — Supplementary Material 4 [file 12955_2024_2289_MOESM4_ESM.docx]

**Additional file 3** COSMIN Risk of Bias overall ratings for content validity

|  | **SNOT-16** | **MARS** | **RhinoQoL** | **PRSS** | **S5** |
| --- | --- | --- | --- | --- | --- |
| **PROM development** | Inadequate  (PROM development not performed in a sample representing the target population) | Inadequate  (PROM development based on literature search) | Inadequate  (PROM development not performed in a sample representing the target population) | Doubtful  (PROM development based on a quantitative survey; inappropriate sample size) | Inadequate  (PROM development not performed in a sample representing the target population) |
| **Content validity** | Not performed | Not performed | Not performed | Not performed | Not performed |

*MARS* Measurement of Acute Rhinosinusitis, *PRSS* Pediatric Rhinosinusitis Symptom Score, *RhinoQoL* Rhinosinusitis Quality-of-Life Questionnaire, *SNOT-16* Sinonasal Outcome Test-16, *S5* Sinusitis Symptom Questionnaire
